# The Socioeconomic Benefit to Individuals of Achieving the 2020 Targets for Five Preventive Chemotherapy Neglected Tropical Diseases

**DOI:** 10.1371/journal.pntd.0005289

**Published:** 2017-01-19

**Authors:** William K. Redekop, Edeltraud J. Lenk, Marianne Luyendijk, Christopher Fitzpatrick, Louis Niessen, Wilma A. Stolk, Fabrizio Tediosi, Adriana J. Rijnsburger, Roel Bakker, Jan A. C. Hontelez, Jan H. Richardus, Julie Jacobson, Sake J. de Vlas, Johan L. Severens

**Affiliations:** 1 Institute of Health Policy & Management, Erasmus University Rotterdam, Rotterdam, The Netherlands; 2 Neglected Tropical Diseases, World Health Organization, Geneva, Switzerland; 3 Centre for Applied Health Research and Delivery, Department of International Public Health, Liverpool School of Tropical Medicine and University of Liverpool, Liverpool, United Kingdom; 4 Department of Public Health, Erasmus MC, University Medical Center Rotterdam, Rotterdam, The Netherlands; 5 Swiss Tropical and Public Health Institute, University of Basel, Basel, Switzerland; 6 Medical Delta, Delft, The Netherlands; 7 Bill & Melinda Gates Foundation, Seattle, WA, United States of America; George Washington University School of Medicine and Health Sciences, UNITED STATES

## Abstract

**Background:**

Lymphatic filariasis (LF), onchocerciasis, schistosomiasis, soil-transmitted helminths (STH) and trachoma represent the five most prevalent neglected tropical diseases (NTDs). They can be controlled or eliminated by means of safe and cost-effective interventions delivered through programs of Mass Drug Administration (MDA)—also named Preventive Chemotherapy (PCT). The WHO defined targets for NTD control/elimination by 2020, reinforced by the 2012 London Declaration, which, if achieved, would result in dramatic health gains. We estimated the potential economic benefit of achieving these targets, focusing specifically on productivity and out-of-pocket payments.

**Methods:**

Productivity loss was calculated by combining disease frequency with productivity loss from the disease, from the perspective of affected individuals. Productivity gain was calculated by deducting the total loss expected in the target achievement scenario from the loss in a counterfactual scenario where it was assumed the pre-intervention situation in 1990 regarding NTDs would continue unabated until 2030. Economic benefits from out-of-pocket payments (OPPs) were calculated similarly. Benefits are reported in 2005 US$ (purchasing power parity-adjusted and discounted at 3% per annum from 2010). Sensitivity analyses were used to assess the influence of changes in input parameters.

**Results:**

The economic benefit from productivity gain was estimated to be I$251 billion in 2011–2020 and I$313 billion in 2021–2030, considerably greater than the total OPPs averted of I$0.72 billion and I$0.96 billion in the same periods. The net benefit is expected to be US$ 27.4 and US$ 42.8 for every dollar invested during the same periods. Impact varies between NTDs and regions, since it is determined by disease prevalence and extent of disease-related productivity loss.

**Conclusion:**

Achieving the PCT-NTD targets for 2020 will yield significant economic benefits to affected individuals. Despite large uncertainty, these benefits far exceed the investment required by governments and their development partners within all reasonable scenarios. Given the concentration of the NTDs among the poorest households, these investments represent good value for money in efforts to share the world’s prosperity and reduce inequity.

## Author Summary

The five most prevalent neglected tropical diseases (NTDs) are lymphatic filariasis (LF), onchocerciasis, schistosomiasis, soil-transmitted helminths (STH) and trachoma. They can be controlled or eliminated by means of safe and cost-effective interventions delivered through programs of Mass Drug Administration (MDA)—also named Preventive Chemotherapy (PCT).The WHO defined targets for NTD control/elimination by 2020, reinforced by the 2012 London Declaration, which, if achieved, would result in benefits not limited to health gains. We estimated the potential economic benefit of achieving these targets for these NTDs. Economic benefit was calculated by combining the estimated disease frequency with productivity loss resulting from the disease, from the perspective of a person affected by each of the NTDs. The same was done for the healthcare costs paid by the affected individuals. The economic benefit to individuals from productivity gain was estimated to be I$ 251 billion in 2011–2020 (before achieving the targets) and I$ 313 billion in 2021–2030 (after achieving the targets). The estimated total healthcare costs averted are substantial though lower than productivity costs: $0.72 billion in 2011–2020, $0.96 billion in 2021–2030. This would mean a return of US$ 27.4 for each dollar spent between 2015–2020 to reach the targets and US$ 42.8 between 2021–2030. The economic impact varies between NTDs and regions, since it is determined by disease prevalence and the degree of productivity loss caused by the disease.

## Introduction

Neglected tropical diseases (NTDs) are a group of debilitating infectious diseases that can result in death, but are more often associated with chronic, disabling and disfiguring morbidity [1]. Most of them affect forgotten people, with little political or financial capital, living in slums or in rural areas (predominantly in low–and middle-income countries), away from cities where policymakers live and work. [2–5]

Besides having devastating consequences for one’s health, NTDs also have an important effect on the economic welfare of patients and their families, imposing additional economic difficulties for populations struggling to live below the poverty line of 1US$ a day [4,6–13]. In this sense, NTDs are an additional obstacle to sustainable development, which can be achieved only in the absence of high prevalence of debilitating communicable and non-communicable diseases. [14]

NTD control or elimination targets for the year 2020 were set out in the WHO Roadmap of 2011 and endorsed by partners in the London Declaration of 2012 [15–17]. Among these 10 NTDs, the most prevalent globally are: onchocerciasis, lymphatic filariasis (LF), schistosomiasis, soil-transmitted helminths (STH), and trachoma. [9,18,19] Even though they can be controlled or eliminated by means of safe and cost-effective interventions delivered through programs of Mass Drug Administration (MDA)—also named Preventive Chemotherapy (PCT)—NTD programs still face many obstacles [9]. Some of them are closely related to the degree of awareness that policymakers and the global health communities have of the health and socioeconomic importance of NTDs [13]. To bring more light onto this issue, de Vlas et al. estimated the health gains of achieving the 2020 targets. [16,17]. Their findings suggest that roughly 300 million disability-adjusted life-years (DALYs) can be averted for the five PCT-NTDs over the period 2010–2030. [20]

An improved understanding of the economic impact that NTDs have on individuals, households, and countries would help in informing the scale-up of effective and equitable interventions to address them [8]. Therefore, robust and clear estimates of the cost of NTDs to patients, households and broader societal costs are needed. Combining these cost estimates with the already existing estimates of disease burden and comparing the result to the estimated cost of intervention could bolster the case for increased investment.

The aim of the study described in this article was to estimate the socioeconomic benefit (to individuals) of controlling or eliminating PCT-NTDs. More specifically, we examined the productivity loss and out-of-pocket payments (OPPs) that can be prevented globally, assuming the 2020 WHO targets for these diseases will be met.

## Methods

### Study design

The estimate of economic benefit was based on the health benefit calculated by De Vlas et al.[20] Briefly, De Vlas et al used 2010 GBD data of prevalent cases of the NTDs included in the London Declaration for the years 1990 and 2010 as the starting point in their calculations, with estimates for other years obtained by interpolating between 1990 and 2010, and further extrapolated until 2030, under the assumption that the 2020 WHO Roadmap targets were met and sustained beyond 2020. They first created a “counterfactual scenario”, which assumed that the epidemiological situation from 1990 regarding NTDs would continue unabated and that the number of cases would increase as a function of overall population growth. They estimated the numbers of disease cases that would be expected if the 2020 targets mentioned by the 2012 London Declaration and described by the WHO were to be achieved (target achievement scenario). Disease cases averted by achieving the targets were calculated for each GBD disease manifestation (or “sequela”), country, sex and age group. For a complete list of the GBD sequela per NTD, refer to Salomon et al (2012).[21] Guinea worm (dracunculiasis) was not included in this study, since it was targeted to eradication in 2015. [22]

De Vlas et al. used a time period of 2011–2030 that extended beyond 2020 since some of the benefits of achieving the targets will only arise after that year. That is, the benefits of preventing permanent sequelae like blindness will appear much later than the benefits of better treatment of other sequelae. [20]

### General approach to estimate the economic benefits

This economic study builds on the approach used by de Vlas et al. to assess the health benefits of achieving the 2020 targets. Namely, for each GBD disease sequela a comparison is made between the counterfactual scenario and the target achievement scenario, calculating the benefit for the period between 2011 and 2030 (ten years before and then years after the target achievement) instead of the entire period from 1990 to 2030. The economic benefit was calculated by subtracting the costs calculated for the target achievement scenario from the costs of the counterfactual scenario. [20]

All economic benefit estimates were expressed in international dollars (constant 2005 I$), a hypothetical unit of currency that has the same purchasing power as the U.S. dollar has in the United States at a given point in time (in this case, 2005). It is calculated using purchasing power parity (PPP) exchange rate, defined as the amount of a country's currency required to buy the same amounts of goods and services in the domestic market as U.S. dollar would buy in the United States. It is regarded as a more valid measure to compare estimates between countries. [23,24]

Economic benefits from productivity gain and out-of-pocket payments were reported separately, as recommended by the WHO. Intangible costs and leisure time were not included.[25–27] Discounting at 3% was applied using the base year of 2010. All calculations were performed using Microsoft Excel (version 2010). [28]

#### Time frame

The estimated economic benefits are presented for 2011–2020 and 2021–2030 separately to show how much of the benefits can be expected before the 2020 targets are met compared to after 2020.

#### Perspective

We used a microeconomic perspective to analyze the economic costs per GBD sequela, sex and country. Only the most important costs that individuals in low- and middle-income countries incur during illness were included in the analysis, namely indirect costs due to lost productivity and the direct costs of obtaining health goods and services [25].

#### Countries

The countries included in the analysis were the ones with disease cases for each sequela according to the GBD study. The list of countries differs according to disease and sequela. The list of countries per disease can be accessed by using the open-access web-based dissemination tool available here: https://erasmusmcmgz.shinyapps.io/dissemination/. [20]

### Productivity loss

**Fig 1:**
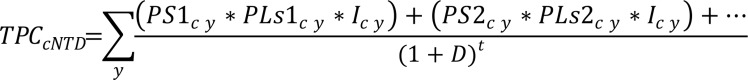
General formula for calculating productivity loss. TPC = Total productivity costs (in US$ 2005)NTD = Neglected Tropical Diseasec = Countryy = YearPS1 = Number of prevalent cases aged 15+ years with sequela 1PS2 = Number of prevalent cases aged 15+ years with sequela 2PLs1 = % productivity loss related to sequela 1 of NTDPLs2 = % productivity loss related to sequela 2 of NTDI = GDP per capita in the lowest quintileD = Annual discount rate (%)t = Time (years beyond 2010).

The annual productivity loss per NTD was calculated using the formula shown below (Fig 1). The costs per country and NTD were calculated independently for both counterfactual and target achievement scenarios. The economic benefit was obtained by comparing the target achievement scenario to the counterfactual scenario. Global and region-specific benefits were estimated by adding up the benefits of all countries or the countries within a specific region.

#### Prevalent cases

The number of prevalent cases refers to the average number of cases per country, age group, and sex with a specific disease sequela in any particular moment in a year. The numbers of disease cases of each year in the period 2011–2030 for both the counterfactual and the target achievement scenarios were calculated by de Vlas et al. and used in our analyses [20].

Only individuals older than 15 years were included in the calculation of productivity loss. The lower age limit of 15 was chosen since it is often used by the International Labour Organization (ILO) [29], even though we know that a significant percentage of children aged 5–14 years works in developing countries (sometimes as high as 27%), especially in rural settings. [30,31] All cases older than 65 years were included in the calculations, assuming they would continue to work after this age, since only a small proportion of people effectively receive a pension in developing countries (median: 7%); people living below the US$ 2/day poverty line are even considered not to have any effective basic social protection. [32,33].

#### Productivity loss associated with disease

Productivity loss refers to the amount of time spent on economic activity that is lost due to a specific disease sequela. Economic activity includes time spent in the formal labor market but also time spent on self-sufficient farming, time spent on domestic chores or other unpaid activities. [34,35]

Estimates of the productive time lost due to a specific NTD sequela were not easily obtained, since published literature of population-based studies on this topic is scarce. [36] To address this issue, previous studies have used disability weights as a proxy for extent of productivity loss, assuming a linear relationship between productivity and the disability weight [37,38]. However, there are many reasons to believe that disability weights are not appropriate indicators for productivity loss, since a variety of different health states have almost the same disability weights even though they may result in differing degrees of productivity loss. For example, in the most recent set of disability weights (GBD 2010), blindness has a weight of 0.195, severe anemia has a weight of 0.164 and disfigurement (level 2 with itch or pain) has a weight of (0.187). [21] It seems rather implausible that these various health states lead to the same amount of productivity loss. Perhaps more importantly, the latest published disability weights from 2010 sometimes differ dramatically from previous values. For instance, the most recent weight for blindness is 0.195, much lower than the previous weight of 0.60 [39].

We therefore performed a comprehensive search of the literature to determine the most appropriate estimates of productivity loss related to NTDs. [36] Results were translated into annual proportions of productivity loss per sequela, assuming 300 working days per year [40]. If several estimates of productivity loss were identified in the literature review, we used the lowest value found as the base-case value to keep the estimates conservative. One exception was lymphatic filariasis, where we chose to use the values reported by an earlier review [40]. If no estimates of productivity losses were available in the literature, assumptions were made based on the productivity loss of similar sequelae caused by other diseases.

Table 1 shows the percentage of annual productivity loss per sequela, which ranges from 0% for sequela like mild onchocerciasis skin disease to 79% for blindness. The table shows additional information for onchocerciasis and trachoma since the GBD study only reported the number of cases per disease sequela and not the number in each level of severity. For example, for vision loss due to onchocerciasis, only the numbers of cases with vision loss were reported and not the numbers with blindness, severe vision loss and moderate vision loss. Therefore, in order to calculate an overall estimate of productivity loss, we had to combine our estimates of productivity loss per severity level with the estimated frequencies of the different severity levels (i.e., the ‘case mix’). Table 1 therefore shows the productivity loss according to severity and case mix regarding severity for onchocerciasis and trachoma. The minimum and maximum estimates of productivity loss are shown in the sensitivity analysis section.

**Table 1:** Estimates of productivity loss used in the calculations of economic benefit.

Disease & Sequela	Severity	Base case—Annual productivity loss	Sources	Case^1^ Mix	Sources	Remarks	Weighted productivity loss per sequela
**Lymphatic filariasis**							
Lymphedema		16%	[43–50]	N.A.			N.A.
Hydrocele		15%	[44,47,49,51,52]	N.A.			N.A.
**Onchocerciasis**							
Vision loss	Blindness	79%	[53]	29%	^4^	^5^	49.8%
	Severe	38%	Idem[54]	12%	^4^	^5^	49.8%
	Moderate	38%	Idem[54]	59%	^4^	^5^	49.8%
Skin disease	Moderate	10%	[55]	32%	^4^		3.2%
	Mild	0%	Assumption	68%	^4^	^6^	3.2%
**Schistosomiasis**							
Acute episode		0%	[21]	N.A.		^2^	N.A.
Mild diarrhea		3%	[54]	N.A.			N.A.
Hepatomegaly		3%	Idem[54]	N.A.		^7^	N.A.
Dysuria		1.6%	[56]	N.A.		^7^	N.A.
Bladder pathology		1.6%	Idem[54]	N.A.		^7^	N.A.
Hydronephrosis		1.6%	Idem[54]	N.A.		^7^	N.A.
Hematemesis		100%	[54,57,58]	N.A.			N.A.
Ascites		100%	Idem[54]	N.A.			N.A.
Anemia		7%	[59–65]	N.A.		^3^	N.A.
**Soil-transmitted helminths**							
Ascariasis	Infestation	6%	[64,65]	N.A.		^6^	N.A.
Trichuriasis	Infestation	6%	Idem	N.A.		^6^	N.A.
Hookworm	Infestation	6%	Idem	N.A.		^6^	N.A.
Hookworm	Anemia	6%	[59–65]	N.A.		^6^	N.A.
Ascariasis	Mild abdominopelvic problems	0%	[21]	N.A.		^2^	N.A.
Trichuriasis	Mild abdominopelvic problems	0%	Idem	N.A.		^2^	N.A.
Hookworm	Mild abdominopelvic problems	0%	Idem	N.A.		^2^	N.A.
Ascariasis	Severe wasting	0%	[21]	N.A.		^8^	N.A.
Trichuriasis	Severe wasting	0%	Idem	N.A.		^8^	N.A.
Hookworm	Severe wasting	0%	Idem	N.A.		^8^	N.A.
**Trachoma**							
Vision Loss	Blindness	79%	[53]	35%	^4^	^9^	32%
	Severe Visual Impairment	38%	[53]	10%	^4^	^9^	32%
	Moderate Visual Impairment	0%	Assumption	55%	^4^	^9^	32%

1. Case mix represents the distribution of the different degrees of severity within a disease sequela. Since the prevalent case estimates were only available per disease sequela and not severity, productivity loss values of the different degrees of severity were combined with the case mix to calculate a frequency-weighted value of productivity loss for that sequela. For sequelae with only one level of severity, the productivity loss value was applied to all prevalent cases.

2. The lay description used in the GBD study to describe some sequelae indicated that the sequela “did not interfere / did not impose difficulties with daily activities”, therefore productivity loss assumed 0%.

3. Even though productivity loss due to schistosomiasis and STH-related anemia was based on the same studies, the actual degree of productivity loss differed between the diseases. The GBD documentation describes a higher mean hemoglobin loss due to schistosomiasis (2.8 g/L) than the loss due to hookworm (2.08 g/L). Since the literature showed a linear relationship between hemoglobin loss and productivity loss, this proportion was kept in the calculations of the productivity loss due to schistosomiasis and hookworm anemia (higher percentage of productivity loss due to schistosomiasis anemia than to hookworm anemia).[59,62].

4. Case-mix values from the GBD study documentation and from the assumptions used by de Vlas et al.[20].

5. Evans et al. made no distinction between moderate and severe visual impairment. We assumed that Evans considered the productivity loss from low vision a weighted average of moderate and severe impairment and that the distribution of moderately and severely impaired persons was equal to the distribution used in the GBD study.

6. To ensure a conservative estimate.

7. According to the GBD study, the sequela “did not interfere/did not impose difficulties with daily activities”. Since clinical experience and literature have shown that they interfere with daily activities, productivity loss was not assumed to be 0%.

8. GBD data reported only cases amongst children younger than 5 years, which fell outside the scope of our definition of economically active population of 15+ years.

9. Productivity loss estimates were based on the study of onchocerciasis by Evans et al. since no studies of trachoma-related productivity loss were found and since the GBD descriptions of visual impairment from onchocerciasis and trachoma are similar. Average productivity loss from trachoma differed from that of onchocerciasis because of differences in case mix regarding severity. 0% productivity loss was attributed to moderate visual impairment caused by trachoma to ensure a conservative estimate.

Productivity losses associated with long-term cognitive impairment due to helminthic infections and productivity compensation mechanisms were not calculated in our analyses. This is discussed later in the Discussion section.

#### Income

In this study, income refers to annual income losses to patients as a consequence of NTD sequela. As in earlier economic impact studies of NTDs, productivity loss was calculated by using the human capital approach, which uses income to place monetary terms on healthy time [25,40,41]. The human capital approach has been criticized for its use in the estimation of productivity losses experienced by society as a whole, in part because it assumes full employment (i.e. that there are no unemployed individuals to replace the sick ones). [25] We limit our analysis to the productivity loss of affected individuals.

Estimating the income of individuals with NTDs is difficult, since these diseases are mainly prevalent in rural areas where self-sufficient farming is one of the most–and sometimes the most—important sources of revenue [9]. Previous economic analyses of NTDs have applied different methods to estimate the rural wage, including use of GDP per capita, average agricultural value added per worker and the lowest wage estimate from different predefined wage sources [37,38,40].

We compared the GDP per capita of the lowest quintile with the minimum nominal annual wage (both 2010 PPP), for the endemic countries with the highest number of prevalent cases (which would have the highest impact on the final results), showing that the minimum wage would still be higher. Since NTDs are generally known as diseases of the poorest, we decided to use the GDP per capita of the lowest income quintile for the calculations of this study, and use only one data source for income instead of several (this parameter was varied with the lowest decile and the second lowest quintile in the sensitivity analysis). The World Bank Development Indicators website provided the data needed to calculate the average GDP per capita in the different quintiles since this website reports the GDP per capita (PPP) in international dollars of the year 2005 and income shares of the population in the different quintiles [42]. In the rare cases where information about GDP per capita or income shares of the year 2010 for a country was missing, we used data from preceding years; if no information from any year was available, we used the average of surrounding countries. In order to keep estimates conservative, we assumed that income and income shares remained constant over time, and we did not adjust income for labor force participation or age-related income patterns.

### Out-of-pocket payments

**Fig 2:**
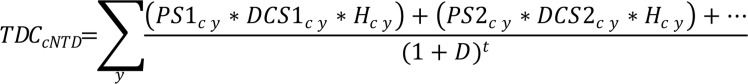
General formula for calculating out-of-pocket payments. TDC = Total Direct Costs (in US$ 2005)NTD = Neglected Tropical Diseasec = Countryy = Year PS1 = Number of prevalent cases with sequela 1PS2 = Number of prevalent cases with sequela 2DCS1 = Annual direct costs sequela 1DCS2 = Annual direct costs sequela 2H = percentage of individuals seeking health careD = Annual discount rate (%)t = Time (years beyond 2010).

We used one general formula to estimate the total out-of-pocket payments (OPPs) (Fig 2). This formula was applied for each country and NTD separately, for both the counterfactual and the target achievement scenarios (Fig 2). The economic benefit was calculated by taking the difference between the results using the target achievement scenario and the results using the counterfactual scenario. Global and region-specific benefits were estimated by adding up the benefits of all countries or the countries within a specific region. In contrast to the estimation of the productivity loss, that was calculated for individuals older than 15 years, OPPs were calculated for all prevalent cases, including children, since these costs are unrelated to the ability to work.

#### Prevalence of NTDs and their sequelae

The same prevalence estimates used to calculate the productivity loss were used to calculate the OPPs.

#### Annual direct costs

Direct costs for health care refer to the costs that arise from seeking treatment to enhance or restore health and are paid for by the patient himself (e.g. payments made to health practitioners or suppliers of pharmaceuticals) [25]. Our literature review showed that relatively few studies have quantified direct costs for the five PCT diseases. This is not surprising considering that many drugs to cure these five diseases are donated for free by several international partnerships. [16,17] In this case, costs to the individual were assumed to be zero (except for LF, as explained below) even though individuals receiving free treatment might bear travel, escort, accommodation, food and other costs themselves. Another reason for the scarcity of information on direct costs is that initial complaints are often not considered important enough to seek health care, and once chronic sequelae have developed, only palliative measures can be taken, for instance onchocerciasis, trachoma, or lymphatic filariasis. [43–50,55]

We assumed that blindness and skin disease due to onchocerciasis do not lead to any substantial out-of-pocket payments. First, there is no treatment for blindness, which is irreversible. Second, in the absence of a control program, skin disease is often not considered important enough for patients to seek health care. [66] We therefore ignored any additional costs since we did not expect any substantial additional costs for skin disease and since no publications describe additional OSD-related OPPs.

Soil-transmitted helminthiasis and schistosomiasis were assumed to be cured with the anti-parasitic medication, resulting in minimal chronic sequelae and costs. [67,68]

Just as with blindness due to onchocerciasis, blindness and low vision due to trachoma were assumed not to lead to any OPPs. This is not to say that no OPPs are expected, since previous studies have shown that patients with trichiasis can be treated surgically, which can result in additional spending for patients despite the fact that surgery is often provided for free.[69–74] However, since prevalence estimates of trichiasis were not included in the GBD database, trichiasis-related OPPs (and productivity costs) could not be included in this study.

Previous studies on lymphatic filariasis have shown that the expenditures of patients seeking treatment due to LF sequelae are not negligible, even after they are treated with anti-parasitic drugs. [40,43,44,46,47,52,75–83] Therefore, we calculated the direct costs arising from lymphedema and hydrocele. Annual out-of-pocket payment costs were calculated for each WHO region separately since treatment type and costs can vary between regions. Table 2 shows the values used to estimate the OPPs for LF, which were kept constant over time. These values, used in the study by Chu et al., included costs for medicines, consultation fees, transport, food, accommodation and others. They were not only divided according to sequela, but also by WHO region, with the exception of India, for which country-specific estimates were available. [40]

**Table 2:** Direct costs used for lymphatic filariasis with lower and upper limits [between brackets].

LF Sequela	WHO region	Annual Direct Costs^2^ (int US$)	Patients Seeking Treatment	Patients that have ADLA	Source
ADLA^1^ Lymphedema	WHO AFRO^3^	0.36 [0.6–1.25]	65% [55% - 70%]	95% [90–95%]	[40]
	WHO SEARO^4^	5.60 [0.93–19.43]			
	WHO WPRO^5^	19.60 [3.27–68.1]			
	WHO AMRO^6^	6.00^9^ [1.0–20.82]			
	WHO EMRO^7^	0.36 [0.06–1.25]			
	All GPELF countries^8^	6.00 [1.0–20.82]			
	India^11^	(same as SEARO)	85% [65% - 98%]	95% [90–95%]	[40,43,44,80]
ADLA Hydrocele	WHO AFRO	0.18 [0.3–0.62]	65% [55% - 70%]	70% [45% - 90%]	[40]
	WHO SEARO	2.80 [0.47–9.72]			
	WHO WPRO	9.80 [1.63–34.01]			
	WHO AMRO	0.18 [0.03–0.62]			
	WHO EMRO	0.18 [0.03–0.62]			
	All GPELF countries	3.00 [0.50–10.41]			
	India^11^	(same as SEARO)	85% [65% - 98%]	70% [45% - 90%]	[40,43,44,80]
Chronic Lymphedema	All regions	4.3 [0.85–15.0]	50% [30–55%]	-	[40]
	India^11^		65% [49–74%]	-	[40,43,80]
Chronic Hydrocele ^10^	All regions	2.9 [0.55–10.05]	40% [20–50%]	-	[40]
	India^11^		60% [49–74%]	-	

1. ADLA—acute dermatolymphangioadenitis.

2. Based on an average of two ADLA episodes a year for hydrocele and four ADLA episodes a year for lymphedema [40].

3. WHO AFRO—World Health Organization African Region.

4. WHO SEARO—World Health Organization Region South-East Asia.

5. WHO WPRO—World Health Organization Western Pacific Region.

6. WHO AMRO—World Health Organization Americas Region.

7. WHO EMRO—World Health Organization Eastern Mediterranean Region.

8. GPELF–Global Programme to Eliminate Lymphatic Filariasis.

9. Since the region-specific estimate was not mentioned, the global average was used [40].

10. Hydrocelectomy already included in the chronic cost of hydrocele.

11. Different estimates were used for India due to more primary data available suggesting estimates differ from other regions. [40].

#### Percentage of patients seeking care

Percentage of patients seeking care refers to the percentage of patients who seek treatment for that NTD sequela (shown in Table 2).

### Productivity gain of premature mortality averted

The number of productive years lost due to NTD-related premature mortality was estimated using country-, age-, and sex-specific mortality data for every year in the 1990–2030 period. The number of lost work-years was determined for the two scenarios (counterfactual scenario and target achievement scenario). The productive years lost per person were estimated by comparing the year in which the person died due to an NTD (e.g., ascariasis) and the year in which the person would otherwise have died according to country-specific life expectancies. The difference between these two years reflected the total number of productive years lost due to an NTD. We then restricted these lost productive years to the time period used in our analyses (i.e., 2011–2030). Economic benefit in the 2011–2030 period was calculated by combining the lost productive years with income per person.

### Sensitivity analysis

Economic benefit was calculated by combining various base-case values for disease prevalence, income, and productivity loss. This raised the question of how much the estimate of benefit would change if the values of the different input parameters were to change. Probabilistic sensitivity analyses were therefore performed to determine how much the statistical uncertainty about the values of input parameters influenced the estimated economic impact. In these analyses the values of three input parameters were allowed to vary simultaneously, assuming that there was no correlation between the values of the different parameters.

The input parameters in our analysis included: 1) the GBD estimates of disease prevalence in 2010; 2) percentage of productivity loss and OPP per individual, and 3) income. The values of these parameters were varied using a beta PERT distribution in combination with the point estimate and the upper and lower limits for each input parameter. The limits used were based on different data sources or unavoidable assumptions. Table 3 shows the upper and lower limits used for the uncertainty regarding prevalence, productivity loss and income, per disease, while Table 2 shows the upper and lower limits used for the calculations related to OPPs.

**Table 3:** Lower and upper limits used in the sensitivity analyses.

Relative uncertainty in global prevalence in 2010				Estimates of productivity loss ^1^			Estimates of income		
	Lower limit	Point estimate	Upper limit	Lower limit	Point estimate	Upper limit	Lower limit	Point estimate	Upper limit
Lymphatic filariasis	0.886	1.000	1.136	10%	15%	20%	0.871	1.000	1.424
Onchocerciasis	0.936	1.000	1.103	14%	17%	30%	0.836	1.000	1.673
Schistosomiasis	0.987	1.000	1.027	2.5%	4%	18%	0.815	1.000	2.352
STH (anemia)	0.861	1.000	1.228	3%	6%	12%	0.766	1.000	2.106
STH (infestation)				3%	6%	9%			
Trachoma	0.694	1.000	1.421	16%	32%	63%	0.871	1.000	1.424

1. The productivity loss estimates seen in Table 1 are here shown as frequency-weighted estimates per sequela and per disease with their respective upper and lower limits used in the sensitivity analysis.

The importance of uncertainty about the prevalence in 2010 and the years thereafter was examined by applying the country-specific upper and lower confidence intervals of the GBD estimates for 2010 to all the years in the 2010–2030 period.

Uncertainty about the actual productivity loss and OPP from having one of the five diseases was addressed by using the highest and lowest values from studies with sufficient quality found through the literature review and estimating a frequency-weighted estimate of productivity loss and OPP per disease.

The variation in the income estimates was based on the variation of income of the country with the highest number of prevalent cases for each disease. The lower limit was the average income in the lowest decile, while the upper limit was the average income in the second-highest quintile of these countries. The ratio between the income in the lowest quintile (point estimate) and the income in the lowest decile and second-highest quintile (limits) in these countries was then applied to the general income variation.

Since the sensitivity analysis was performed varying these three variables simultaneously, the actual lower and upper limits in the sensitivity analysis were broader than the individual ones shown by Table 3.

### Return on investment

We calculated a rough estimate of the net return on investment (ROI) from 1990 to 2020 (NTD Roadmap targets) and to 2030 (the SDG target). Net ROI is the present value of the benefit to affected individuals minus the present value of the cost to public and philanthropic funders, divided by the present value of the cost to public and philanthropic funders. We used the economic benefit to affected individuals of averted OPP and productivity loss calculated in this study, and the investment costs based on recent WHO estimates from the Third Report on Neglected Tropical Diseases. [22]

We converted the I$ 2010 benefits to US$ 2015, for direct comparison to the investment targets. Evidently, part of these benefits is attributable to investments made before 2011; in calculating benefits net of costs we therefore had to estimate investments in the period 1990–2010. We conservatively assumed that investments in the period 1990–2010 were at the same level of those in 2011 (in real terms). 1990 is assumed to mark the beginning of concerted global efforts to control most NTDs and 2011 is assumed to mark the beginning of the recent scale-up in investment to eliminate them. In reality, investments before 2011 were probably lower than this in most countries. Investments in improving housing and water and sanitation that occurred over the same period were not considered, since these were not targeted at the NTDs but nonetheless contributed to their control. We did not estimate the ROI for middle and low income settings separately due to lack of the necessary data on investments. We applied a discount rate of 3% per annum for both costs and benefits. [84]

## Results

### Timing of economic benefits

**Fig 3:**
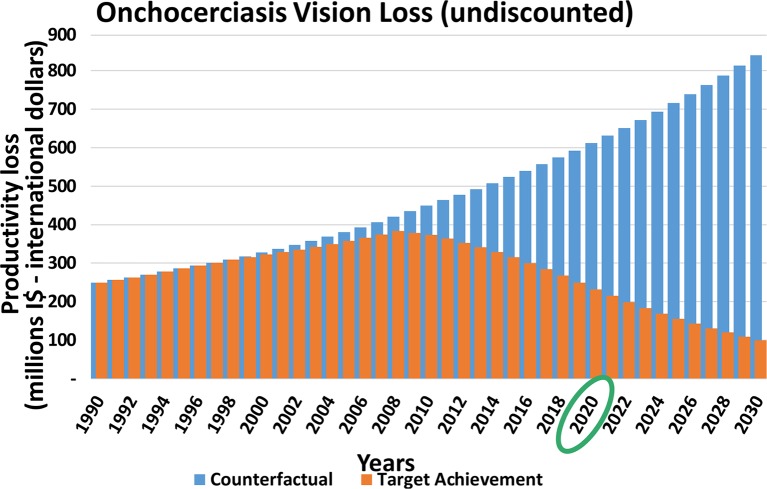
Productivity loss due to skin disease from onchocerciasis according to the counterfactual and target achievement scenarios (millions I$—international dollars) Total global loss per year in the counterfactual scenario (blue) and target achievement scenario (orange). The economic benefit is the difference between both scenarios.

Fig 3 shows the pattern of the global productivity loss over time (1990–2030) of onchocerciasis skin disease. Onchocerciasis skin disease serves as an example; however for other diseases similar patterns can be seen.

The blue bars in Fig 3 represent the total global loss per year in the counterfactual scenario. The increase in loss over time is simply a result of population growth. The orange bars represent the global loss in the target achievement scenario, which gradually reduces over time. The difference between the blue and the orange bars is the economic benefit.

As a result of fewer cases after the targets are achieved in 2020, the benefits for the period 2021–2030 will be higher than for the period 2011–2020 (Table 4). For some disease sequelae the differences between the two periods will be significant (e.g. trachoma-related blindness) whereas for other diseases the difference is small (e.g. onchocerciasis skin disease). This can partly be explained by the differences in the targets between the diseases, which will affect the numbers of cases expected in 2020 when the targets are achieved. [16,17] More importantly, some disease sequelae are reversible whereas others are irreversible. Reversible sequelae can be cured or prevented (e.g. skin disease) whereas irreversible sequelae (e.g. blindness) can only be prevented. As a result, it is expected that the number of patients with reversible sequelae will decrease quickly and few patients will persist after 2020. In contrast, the prevalence of irreversible sequelae will decline more gradually and will persist for many years after 2020.

**Table 4:** Total economic benefit from productivity loss averted, base case estimates and 2.5^th^ and 97.5^th^ percentiles (billions I$—international dollars and US$—US dollars, discounting 3% from 2010).

Disease	Sequelae	Base Case Estimates I$—International dollars		Base Case Estimates US$—US dollars	
		2011–2020	2021–2030	2011–2020	2021–2030
**Lymphatic filariasis**	Lymphedema	$ 12.7	$ 16.7	$ 4.4	$ 5.9
	Hydrocele	$ 18.1	$ 22.8	$ 6.1	$ 7.9
	Total	$ 30.8 [22.6–41.1]^1^	$ 39.5 [28.9–52.8]^1^	$ 10.5 [7.7–14.0]^1^	13.8 [10.1–18.4]^1^
**Onchocerciasis**	Skin disease	$ 0.68	$ 0.86	$ 0.32	$ 0.41
	Vision loss	$ 1.9	$ 3.6	$ 0.87	$ 1.7
	Total	$ 2.6 [1.9–4.0]^1^	$ 4.4 [3.2–6.9]^1^	$ 1.19 [0.88–1.84]^1^	$ 2.11 [1.52–3.27]^1^
**Schistosomiasis**	Anemia	$ 8.7	$ 17.7	$ 3.7	$ 7.5
	Ascites	$ 0.38	$ 1.4	$ 0.2	$ 0.7
	Bladder pathology	$ 0.17	$ 0.63	$ 0.1	$ 0.3
	Dysuria	$ 0.62	$ 1.5	$ 0.3	$ 0.7
	Hematemesis	$ 0.18	$ 0.66	$ 0.1	$ 0.3
	Hepatomegaly	$ 0.96	$ 2.3	$ 0.4	$ 1.1
	Hydronephrosis	$ 0.56	$ 1.37	$ 0.3	$ 0.6
	Mild diarrhea	$ 0.6 ^2^	$ 1.5 ^2^	$ 0.3 ^2^	$ 0.7 ^2^
	Schistosomiasis deaths	$ 0.85	$ 1.7	$ 0.37	$ 0.74
	Total	$ 12.4 [6.2–35.0]^1^	$ 27.4 [13.6–77.2]^1^	$ 5.5 [2.7–15.4]^1^	$ 11.9 [5.9–33.7]^1^
**STH**	Ascariasis deaths	$ 0.03	$ 0.1	$ 0.01	$ 0.04
	Ascariasis infestation	$ 47.1	$ 43.5	$ 19.8	$ 18.2
	Hookworm anemia	$ 116.8	$ 142.8	$ 48.3	$ 58.7
	Hookworm infestation	$ 25.1	$ 28.0	$ 10.4	$ 11.5
	Trichuriasis infestation	$ 13.6	$ 16.5	$ 5.9	$ 7.3
	Total	$ 202.8 [141.0–303.4]^1^	$ 231.0 [160.5–345.6]^1^	$ 84.4 [58.7–126.4]^1^	95.7 [66.6–143.4]^1^
**Trachoma**	Vision loss	$ 1.9	$ 10.4	$ 0.71	$ 3.6
	Total	$ 1.9 [1.0–3.3]	$ 10.4 [5.5–17.8]	$ 0.71 [0.37–1.23]^1^	$ 3.6 [1.25–6.16]^1^
**Total (all diseases)**	** **	**$ 250.6 [186.6–346.3]**^**1**^	**$ 312.8 [233.0–432.4]**^**1**^	**$ 102.3 [76.2–141.4]**^**1**^	**127.2 [86.1–203.4]**^**1**^

1. Sensitivity analyses’ 2.5^th^ and 97.5^th^ percentiles between brackets.

2. Reported in millions International (I$) and US Dollars (US$) due to the comparatively small numbers.

### Total global overview of the economic benefit from averted productivity loss

As a result of achieving the London Declaration targets for the five PCT diseases many individuals will be cured or prevented from having one of these diseases. This can lead to economic benefits for individuals by averting direct treatment costs in terms of OPPs and indirect productivity losses. This can result in billions of dollars of benefit per disease on a global scale, with a total of I$ 250.6 billion (US$ 102 billion) productivity costs averted for the period 2011–2020 and I$ 312.8 billion (US$ 127 billion) for 2021–2030 for the five PCT diseases together (Table 4). The 2.5^th^ and 97.5^th^ percentile values calculated through the sensitivity analyses are I$ 186.6–346.3 billion and I$ 233.0–432.4 billion respectively (US$ 76.2–141.4 billion and US$ 86.1–203.4 billion), for the same periods.

**Fig 4:**
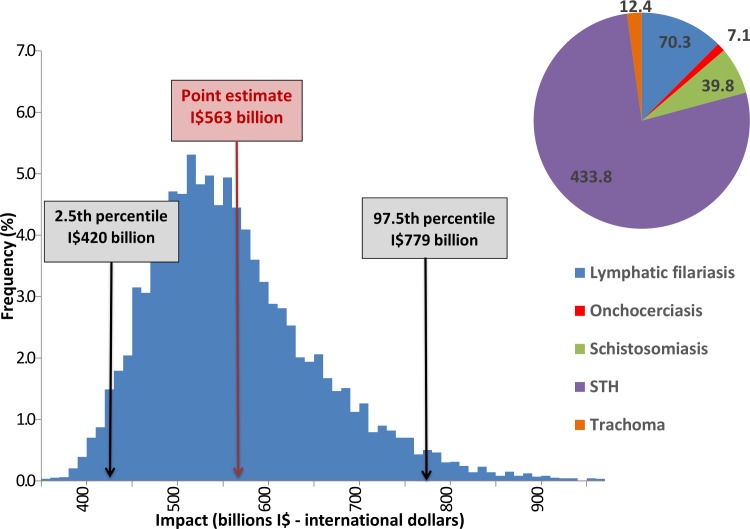
Global economic benefit (productivity loss prevented) for the period 2011–2030 (billions I$—international dollars) Global economic benefit from reaching the targets for 5 PCT diseases, lower and upper estimates from sensitivity analysis.

Table 4 shows the global base case economic gain per disease sequela, for the periods 2011–2020 (before target achievement) and 2021–2030 (after target achievement) and the totals, with the 2.5^th^ and 97.5^th^ percentile calculated in the sensitivity analysis. Fig 4 shows the total values per disease (in I$) together with the sensitivity analysis diagram of the calculations of the total economic benefit of achieving the 2020 targets for the PCT diseases.

**Fig 5:**
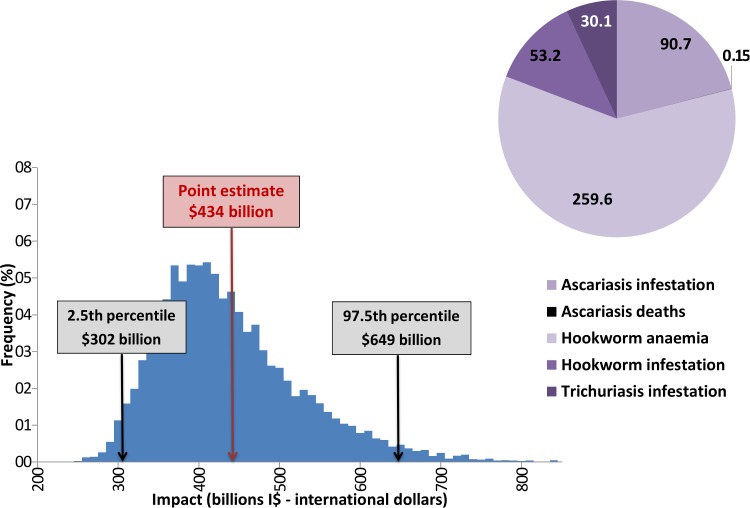
Global economic benefit or reaching the targets for STH (point estimates), per disease sequela for the period 2011–2030 (billions I$—international dollars) STH is the disease responsible for the largest economic impact, especially the anemia sequela. The 2.5 and 97.5 percentiles calculated in the sensitivity analysis are shown in the diagram.

The disease that is responsible for the largest economic benefits is clearly STH, especially the anemia sequela (Figs 4 and 5), accounting for almost 60% of the benefits from reaching the targets for STH. Even though it causes a relatively low productivity loss, this finding can be explained by the widespread distribution of STH.

**Fig 6:**
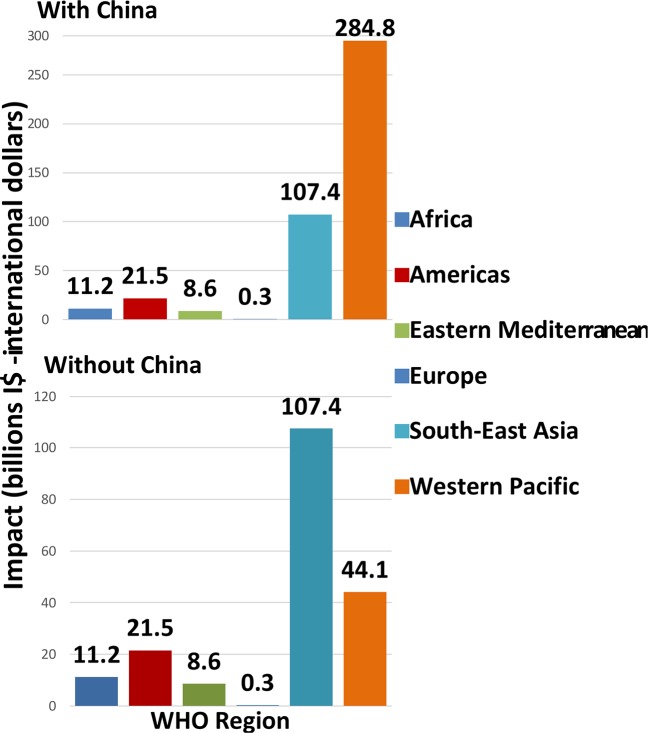
Economic benefit of reaching the PCT targets per WHO region, with and without China Regional variation in the economic benefit, per WHO region, where the Western Pacific region outweighs the benefits of all the other regions when China is included, mainly due to the control of STH. The South East Asia region has the highest benefits when China is not included, due to the impact in India.

Fig 6 highlights the regional variation in the economic benefit, per WHO region. [85] Unsurprisingly, when China is included, the Western Pacific region clearly outweighs the benefits of all the other regions, mainly due to the control of STH. The South East Asia region has the highest benefits when China is not included, which is due to the impact in India.

### Out-of-pocket payments versus productivity loss

Economic benefit due to prevention of lost working time is considerably higher compared to the benefit due to the prevention of direct OPP (I$ 31 billion vs I$ 0.72 billion in 2011–2020 and I$ 40 billion vs I$ 0.96 billion in 2021–2030, corresponding to US$ 11 billion vs US$ 0.25 billion and US$ 14 billion vs US$ 0.33 billion in the same periods). This is attributable to relatively inexpensive medicines compared to income.

### Economic benefit of averted premature mortality

The economic benefit from averted premature mortality was rather small compared to the benefit of averted morbidity, amounting to just 0.48% of the total economic benefit for the period 2011–2030. Within the diseases that presented death cases, it corresponded to 0.03% of the STH benefit and 6.41% of the schistosomiasis benefit.

### Return on investment

The ROI was calculated considering a benefit to individuals of US$ 119.7 billion in the period 2015–2020 and US$ 399 billion in the period 2015–2030, if the 2020 targets for PCT diseases were to be met and considering costs to funders of US$ 2.8 billion and US$ 6.2 billion in the same periods.

The net benefit is US$ 27.4 for every dollar invested during the period 1990–2020 and US$ 42.8 for every dollar invested in the period 1990–2030 (best estimates). More detailed information on the ROI and the internal rate of return per WHO region, as well as other considerations on the investment case of ending/controlling NTD can be found in a forthcoming publication. [84]

The health benefits calculated by De Vlas et al and the economic benefits shown here will be publicly available through the open access website: https://erasmusmcmgz.shinyapps.io/dissemination/.

## Discussion

This is the first study estimating the global economic benefits of achieving WHO Roadmap targets endorsed by the London Declaration for the five PCT diseases. Prevalence estimates based on the GBD study, productivity loss and OPP values based on published literature and income estimates based on World Development Indicators data were used. The productivity costs were calculated using the human capital approach and reported separately from the OPPs [25]. Averted costs were calculated by comparing expected costs of the target achievement scenario with a counterfactual scenario.

Our findings suggest that by averting the five PCT disease, a total of I$ 563 billion (US$ 229 billion) of productivity loss and I$1.7 billion (US$ 0.58 billion) OPPs can be averted over the 20 year time period 2010–2030. This implies that roughly I$ 28 billion (US$ 11 billion) on productivity loss only can be averted annually.

### General approach

We used the human capital approach to calculate the productivity loss. Although this method is generally used and therefore increases comparability with previous studies, some would argue that it overestimates the actual productivity loss from the societal perspective [86]. Since we focused on the economic loss to individuals affected by NTDs, the alternative approach of the friction cost method (which focuses only on the lost productivity until a replacement can be found) was not considered. The consequences of the five PCT diseases are often chronic in nature, which mostly leads to reduced productivity while working (presenteeism) rather than lost work days (absenteeism). For instance, someone with itching due to onchocerciasis will still be able to work but will be less productive at work due to the distracting itching and constant scratching, or a tea plucker with hookworm anemia will not be absent from work, but will earn less due to fewer kilograms of tea plucked. [87,88] While these workers could also be replaced with healthier workers, this is less likely since they are still showing up for work. Therefore, use of the friction method is not expected to have a substantial effect on the estimated economic benefit. This means that the aggregation of individual costs does not exactly represent the total societal cost, since individuals’ productivity losses are still compensated by a variety of mechanisms in the societal level. Some of these mechanisms, for instance household coping strategies and work compensation will be discussed later in this section.

The link between income and productivity depends on more factors than only labor. In the systematic literature review we performed for this study [36], most publications reported productivity in terms of days/hours of work although some publications reported it in terms of output (as kilograms of tea plucked a day, or square meters of constructed road a day). A linear relationship between health and productivity was not always seen and most of the time there was not enough information on this issue. Therefore, and also because of using conservative values for productivity loss, we assumed that average productivity gain would equal marginal productivity gain. The lack of information in the literature prevents us from knowing if this would have under- or overestimated the results.

The impact of NTDs on productivity amongst agricultural families is worth commenting on in more detail since NTDs can lead to productivity loss in different ways. To start with, affected families might own less land than unaffected families as a direct result of NTD-related productivity loss. Oladepo et al reported that ‘farmers with OSD (Onchocercal Skin Disease) had significantly less farmland under cultivation (9,117 m^2^) than those with no OSD (13,850 m^2^)’. But even with a fixed amount of land, healthy farmers could be able to increase their productivity in different ways, including: harvesting more than once a year (enabling them to quickly harvest one crop and plant another); investing more energy in site preparation, which requires more physical strength (e.g., to remove stones and dead trees); working in steep areas that would otherwise be underutilized; and being capable of investing more energy in ameliorating compaction, aeration, soil moisture status, and weeding. It is worth noting that land preparation is by far the most time-consuming activity for the farmer and family, and that weeding accounts for more than 60 percent of the time a peasant farmer spends on the land. [8–92]

Household coping strategies and social security were not included in the calculations. However, household coping strategies can have several effects on the total costs that the illness of a family member can cause for the household. Coping strategies can reduce the impact of lost productivity because another household member can take over the work. For the individual patient these costs are still onerous and may lead to overwhelming effects. Patients may need to borrow money to pay for the treatment or forgo treatment if they cannot afford it. [40] On the other hand, when, for example, children step in and take over the work of their ill parents, they cannot go to school and may therefore have more limited career opportunities later in life. [25]

Productivity loss due to subtle morbidities (for schistosomiasis and STH) as well as productivity loss by informal care were not included in this analysis. The reason for not including the subtle morbidities is that these are not part of the GBD study. However, previous research has shown that subtle morbidity affects people later in life since they may often become trapped in poverty due to low-salary jobs and poorer school performance. [93–96] The productivity loss of caregivers of the blind due to trachoma or onchocerciasis or the severely affected by lymphedema or hydrocele is also not negligible although very little data is currently available.[97] Excluding the effects of subtle morbidity and informal care on productivity loss leads to an underestimation of the economic benefit.

Large differences in economic benefit can be seen between diseases and countries/regions, since economic benefit clearly depends on disease prevalence in a country, the impact that the disease has on productivity and OPPs and income. Comparisons between diseases can only be made with caution. For one, the data limitations and potential biases in the methodology affect the different diseases differently. Furthermore, when multiple diseases can be treated in one single visit, it does not make sense to look at the economic returns from just one disease at a time. Integrated delivery of medicines and preventive efforts means that these returns are in fact complementary. The economic returns from investments in NTDs also depend on how productivity is valued in monetary terms, but the gains are not restricted to them. Physical and mental health of billions of people will be gained with the control/elimination and eradication of NTDs, and attributing exclusively monetary value to these domains in terms of productivity gain underestimates this much bigger gain, of which increased productivity is only a by-product. Therefore, the economic benefit of controlling NTDs (as calculated here) should not be the only argument driving policymaking.

Productivity loss is often minimized by compensating mechanisms, which from the perspective of the individual could be for instance cancelling or postponing work, working extra hours to compensate, or having colleagues compensate for the lost productivity. [98,99] To our knowledge, there is no description in the literature of compensating mechanisms in Low- and Middle-Income Countries (LMIC), and the ones described for firms in developed countries do not necessarily reflect the reality of agricultural families/workers in developing countries. [100] Also, the more chronic nature of sequelae of NTDs leaves less room for work compensation, and some do not even enable people to work due to their severity, such as hepatomegaly or splenomegaly in schistosomiasis, heart failure in Chagas disease, blindness in onchocerciasis and others. Therefore, we do not know the extent to which work compensating mechanisms would have changed the results, but for the reasons just mentioned, probably the results would not change much. [101–103]

### Technical validity

R scripts were written to construct technical validity/calibration of our Excel calculations. The R scripts are completely independent of the Excel calculations and use the same original data (GBD, UNPOP, GDP, productivity loss) as the Excel sheets (though transformed). The few differences we found led to the improvement of the formulae for some of the diseases and later matching of the results, but the general programming in Excel did not change. R scripts and example Excel sheets can be found in the Supporting Information.

### Data sources

#### Prevalent cases

The numbers of prevalent cases were drawn from the 1990 and 2010 GBD estimates and the calculations made by De Vlas et al. [20] The GBD data on which prevalence estimates were based already contained uncertainty ranges, varying in relative terms from 10% less to 40% more compared to the mean (Table 3), depending on availability of country and disease-specific epidemiological data. [20] This uncertainty increased as the GBD numbers were extrapolated over time to estimate the annual prevalence estimates for the period 2010–2030.

In this sense, the extrapolation of the counterfactual estimates may be questioned for several countries that have experienced rapid and large economic growth since 1990, and consequently have not maintained the same epidemiological situation for NTDs as in 1990, as De Vlas et al. have argued. [20] This would mean that the difference between the prevalent cases of the two scenarios would be somewhat smaller. If we take the disease with the biggest economic impact, STH, we would see that more than half of the STH benefits are gained in China due to the possibly overestimated difference between the two scenarios [104]. Nevertheless, if we present the economic benefits excluding the results for China, we would have a lower but still substantial total productivity gain of I$ 126.1 billion for 2011–2020 and I$ 195.8 billion for 2021–2030.

#### Productivity loss and out-of-pocket payments

Literature on productivity loss and out-of-pocket payments was scarce and not available at all for some diseases. [36] As a result, a general global estimate of productivity loss was used for each sequela, which was sometimes based on only one study. When no OPP or productivity loss values were available, other methods were required to estimate productivity loss (e.g. use studies of other diseases with similar sequelae). The few available studies were also often liable to several types of risk of bias, generating large uncertainty around the productivity loss estimates. [36] For instance, the productivity loss estimate used for four schistosomiasis disease sequelae was derived from one article by Fenwick and Figenschou published in 1972, who assessed the difference in productivity of cane cutters uninfected and infected with *Schistosoma mansoni* in Tanzania. [105] In fact, the values of productivity loss used were based on studies performed in very specific contexts, i.e. with tea pluckers, road workers, rubber tappers, farmers, in different countries. The generalizability of these studies to all persons with an NTD may be limited. First, by measuring the productivity loss in working populations, the results will suffer from the ‘healthy worker effect’, since the productivity loss was measured in affected persons that are still able to work, not considering the ones too sick to work [106]. This would underestimate the productivity loss each disease would cause. Second, the productivity loss estimated in the specific regional and working contexts is not necessarily the same for other professions (more or less strenuous, for instance) or for other contexts. Extrapolation of the data from one specific context to others was done due to lack of data, and, depending on the disease sequela, the profession and the working environment, it might have over- or underestimated the extent of productivity loss.

The same productivity loss estimate was used for men and women, despite the fact that this might not always be the case. First, cultural factors may restrict women’s activities to domestic ones, which differ from farming and other occupations that men might have in the same culture; these differences could lead to different productivity losses. [44,46,48,96,107] Second, when performing the same tasks, women can perform differently from men. [64] Third, the same sequela can affect men and women differently. One example is anemia, which might be more frequent and even more severe in women, aggravated by menstrual and birth blood loss, breastfeeding depletion, [62] but might impact the productivity of men even more, since they have more muscle mass and may perform more strenuous tasks than women and are therefore more affected by less efficient blood oxygen transport. Many of the studies investigating productivity loss due to hookworm anemia in men found higher values (18.7–20%) than the ones investigating women (5.4–6.32%). Others showed a bigger impact of anemia on people who perform heavy work compared to those who do light work, so both the difference in severity and the different nature of the jobs could lead to different estimates in productivity loss due to anemia for both women and men.[59–64,108–110] At the same time, a recent systematic review on the impact of hookworm infection and deworming on anemia did also not report gender differences regarding this subject. [111]

As described in the methods, we assumed out-of-pocket payments for onchocerciasis, schistosomiasis, STH, and trachoma to be zero. We know that there are other costs that individuals would have to bear besides transportation costs, such as food, accommodation, escort, diagnostic investigations or procedures (i.e. laboratory) and treatment. Since we did not have enough information on all of these different costs for all PCT diseases and countries included in this study, we assigned a zero value to the direct costs of PCTs (except for LF, as explained before), which led to a conservative estimate of the OPPs.

#### Income

We used the GDP per capita of the lowest income quintile as a proxy for income for the calculations of this study. Compared to the data sources used by other authors described above, the GDP per capita of the lowest quintile seems to be the most conservative estimate without having to combine multiple data sources for income. For instance, when comparing the GDP per capita of the lowest quintile with the minimum nominal annual wage (both 2010 PPP) we would have $ 1592 versus $3453 for China, and $ 1333 versus $2288 for India. [112,113] One could argue that the lowest quintile can refer either to the share of income or of consumption, and that the lower quintile of the population is likely the recipient of considerable transfers, so only part of the income would be earned, and therefore dependent on the worker's productivity. Hence, since NTDs are generally known as diseases of the poorest, using the GDP per capita of the lowest quintile would still overestimate the annual productivity loss of the affected populations. We therefore allowed income to vary to the GDP per capita of the lowest decile in the sensitivity analysis.

### Comparisons with the literature

To our knowledge only a small number of other studies have assessed economic benefits of preventing or treating NTDs. However, these studies did not include all five PCT diseases but examined one specific NTD per study, and most of them one specific country. Comprehensive global assessments were not identified so far.

Frick et al. examined the economic impact of trachomatous visual loss in the year 2000. They estimated an annual productivity cost of $2.9 billion (US$ 1995). This significantly differs from our findings, with an annual average of $620 million for trachoma in the period 2011–2030. Frick used a productivity loss based on disability weights (0.40 for low vision and 0.60 for blindness). We used 79% for blindness but split low vision into severe visual impairment with 35% productivity loss and moderate visual impairment 0% productivity loss. Since trachomatous moderate impairment (55%) is far more common than severe impairment (10%) this together resulted in a much lower productivity loss for low vision compared to what Frick used, namely 6%.

Chu et al. (2010) examined the economic benefits resulting from a global program to eliminate LF. Chu et al. estimated productivity costs and OPP, but they used a different approach to estimate the benefits. They quantified the clinical manifestations that would be averted, while we used GBD sequelae; the health system, was included in their costs calculations, while our approach was focused on the costs of the disease paid by the individual; they calculated the economic benefit for a fixed cohort population, which led to lifetime economic benefits that cannot be compared with our results for a 20-year period.

### Limitations

The comprehensiveness of this economic analysis is limited by the paucity of country/regional data regarding productivity loss and OPPs related to the different NTDs and their sequelae, as well as the characteristics of the affected populations (e.g. income). In addition, assumptions had to be made regarding the predictions of the prevalent cases of each NTD. Even though sensitivity analyses were performed to estimate lower and upper limits of economic benefit, better knowledge and understanding about the abovementioned parameters would improve the estimates. Further research is needed to derive more accurate measures of productivity loss due to NTDs. Furthermore, studies that provide a more accurate characterization of the affected populations would allow a more realistic calculation of economic benefits, due to better information on their socioeconomic context (i.e., details about income, professions, and type of work performed). Future research should also be performed in as many affected countries as possible to shed more light on the socioeconomic differences between the different affected populations in the different countries and enable the consideration of each particular setting in future economic calculations.

Considering that the 2020 targets for the 10 London Declaration NTDs described by the WHO [16,17] will be met of course implies a natural uncertainty about the future. The actual economic benefit will evidently depend on the extent that each country will reach those targets. In this sense, our results do advocate in favor of directing policies that invest in reaching these goals, but our results cannot help deciding on the instruments to reach them.

### Conclusions

The robust findings of our study show that investing in achieving the 2020 WHO targets for the London Declaration NTDs will certainly result in substantial economic gain. The economic benefit to individuals from productivity gain was estimated to be I$ 251 (I$ 187 –I$ 346) billion in 2011–2020 and I$ 313 (I$ 233 –I$ 432) billion in 2021–2030, corresponding to US$ 102 (US$ 76 –US$141) and US$ 127 (US$ 86 –US$ 203) respectively (2.5^th^ and 97.5^th^ percentiles from sensitivity analyses between brackets). Total OPPs averted were I$0.72 (US$ 0.25) billion and I$0.96 (US$ 0.33) billion in the same periods. The best estimates for the net benefit (return on investment) is US$ 27.4 for every dollar invested during the period 1990–2020 and US$ 42.8 for every dollar invested in the period 1990–2030. The impact varies between NTDs and regions, since it is determined by disease prevalence and productivity loss caused by each disease manifestation.

Although the results of this study should be interpreted with care because of the different factors of uncertainty discussed above, we can conclude that economic benefits to individuals will greatly exceed the investments required in interventions. We hope that these results help advocating in favor of addressing the social and environmental determinants of health, especially for the poor and vulnerable, aiming at more equity, inclusion, productivity and health in societies.

Initiatives for joint collection of better socioeconomic and epidemiological data would enable more accurate and complete estimates, leading to better planning and decision-making.

## Supporting Information

S1 File Excel example of calculations of the counterfactual scenario for Onchocerciasis Skin Disease. (XLSM)

S2 File Excel example of calculations of the remaining case scenario for Onchocerciasis Skin Disease. (XLSM)

S3 File R scripts for the calculations of the counterfactual and remaining case scenarios for Onchocerciasis. (ZIP)

## References

[1] Hotez PJ, Alvarado M, Basanez MG, Bolliger I, Bourne R, Boussinesq M, et al The global burden of disease study 2010: interpretation and implications for the neglected tropical diseases. PLoS Negl Trop Dis 2014 7 24;8(7):e2865 10.1371/journal.pntd.0002865 25058013 PMC4109880

[2] Hotez P. Devastating Global Impact of Neglected Tropical Diseases. Microbe Magazine 2009 8;4(8):366–72.

[3] Hotez PJ. NTDs V.2.0: "Blue-Marble Health"—Neglected Tropical Disease Control and Elimination in a Shifting Health Policy Landscape. Plos Neglected Tropical Diseases 2013 11 21;7(11):e2570 10.1371/journal.pntd.0002570 24278496 PMC3836998

[4] Samuels F, Pose RR. Why neglected tropical diseases matter in reducing poverty. Development Progress 2013;Working paper 03.

[5] Houweling TA, Karim-Kos HE, Kulik MC, Stolk WA, Haagsma JA, Lenk EJ, et al Socioeconomic Inequalities in Neglected Tropical Diseases: A Systematic Review. PLoS Negl Trop Dis 2016 5 12;10(5):e0004546 10.1371/journal.pntd.0004546 27171166 PMC4865383

[6] Hotez P, Ottesen E, Fenwick A, Molyneux D. The neglected tropical diseases: the ancient afflictions of stigma and poverty and the prospects for their control and elimination. Adv Exp Med Biol 2006;582:23–33. 10.1007/0-387-33026-7_3 16802616

[7] The Cochrane Collaboration. Neglected Tropical Diseases. 2010; Available at: http://www.thecochranelibrary.com/details/collection/805883/Neglected-tropical-diseases.html. Accessed Apr/08, 2014.

[8] Conteh L, Engels T, Molyneux DH. Socioeconomic aspects of neglected tropical diseases. The Lancet 2010 1/16–22;375(9710):239–247. 10.1016/S0140-6736(09)61422-7 20109925

[9] Hotez PJ, Molyneux DH, Fenwick A, Kumaresan J, Sachs SE, Sachs JD, et al Control of neglected tropical diseases. N Engl J Med 2007 9 6;357(10):1018–1027. 10.1056/NEJMra064142 17804846

[10] CDC—Centers For Disease Control and Prevention. Neglected Tropical Diseases. 2011; Available at: http://www.cdc.gov/globalhealth/ntd/diseases/index.html. Accessed Apr/08, 2014.

[11] Hotez P. Stigma: The Stealth Weapon of the NTD. PloS Neglected Tropical Diseases 2008 4 30;2(4):e230 10.1371/journal.pntd.0000230 18446206 PMC2322832

[12] King CH, Bertino AM. Asymmetries of poverty: why global burden of disease valuations underestimate the burden of neglected tropical diseases. PLoS Negl Trop Dis 2008;2:e209 10.1371/journal.pntd.0000209 18365036 PMC2267491

[13] Hotez P. One World Health: Neglected Tropical Diseases in a Flat World. Plos Neglected Tropical Diseases 2009 4 28;3(4):e405 10.1371/journal.pntd.0000405 19399165 PMC2668801

[14] United Nations. Sustainable Developmnet Knowledge Platform—Health and Population. Available at: https://sustainabledevelopment.un.org/index.php?menu=1251. Accessed March/20, 2015.

[15] Uniting to Combat Neglected Tropical Diseases. Uniting to Combat NTDs & The London Declaration. Available at: http://unitingtocombatntds.org/uniting-combat-ntds-london-declaration. Accessed Apr/07, 2014.

[16] World Health Organization. London Declaration. 2012; Available at: http://www.who.int/neglected_diseases/London_Declaration_NTDs.pdf. Accessed Apr/08, 2014.

[17] World Health Organization. Accelerating work to overcome the global impact of neglected tropical diseases: a roadmap for implementation. 2012:1-37-Available at: http://www.who.int/neglected_diseases/NTD_RoadMap_2012_Fullversion.pdf.

[18] Imperial College London. 7 most prevalent Neglected Tropical Diseases. Available at: http://www3.imperial.ac.uk/schisto/whatwedo/7ntds. Accessed March/11, 2015.

[19] The Global Network for Neglected Tropical Diseases. The 7 Most Common NTDs. Available at: http://www.globalnetwork.org/neglected-tropical-diseases/fact-sheets. Accessed March/11, 2015.

[20] de Vlas SJ, Stolk WA, le Rutte EA, Hontelez JA, Bakker R, Blok DJ, et al Concerted Efforts to Control or Eliminate Neglected Tropical Diseases: How Much Health Will Be Gained? PLoS Negl Trop Dis 2016 2 18;10(2):e0004386 10.1371/journal.pntd.0004386 26890362 PMC4758649

[21] Salomon JA, Vos T, Hogan DR, Gagnon M, Naghavi M, Mokdad A, et al Common values in assessing health outcomes from disease and injury: disability weights measurement study for the Global Burden of Disease Study 2010. Lancet 2012 12 15;380(9859):2129–2143. 10.1016/S0140-6736(12)61680-8 23245605 PMC10782811

[22] World Health Organization. Investing to overcome the global impact of neglected tropical diseases—Third report on neglected tropical diseases. 2015 February:191 pgs-Available from: http://www.who.int/neglected_diseases/9789241564861/en/.

[23] World Health Organization. Purchasing Power Parity 2005. Available at: http://www.who.int/choice/costs/ppp/en/. Accessed March/22, 2015.

[24] The World Bank. GDP per capita, PPP (current international $). Available at: http://data.worldbank.org/indicator/NY.GDP.PCAP.PP.CD. Accessed March/22, 2015.

[25] World Health Organization. WHO guide to identifying the economic consequences of disease and injury. 2009:132pg.

[26] Claeson M, Griffin T, Johnston M, McLachlan A, Soucat A, Wagstaff, et al Health, nutrition and population. Poverty reduction strategy papers’ sourcebook Washington: World Bank.; 2001 p. 201–30.

[27] McIntyre D, Thiede M, Dahlgren G, Whitehead M. What are the economic consequences for households of illness and of paying for health care in low- and middle-income country contexts? Soc Sci Med 2006 2;62(4):858–865. 10.1016/j.socscimed.2005.07.001 16099574

[28] Microsoft. Excel (Part of Microsoft Office) [software]. 2010.

[29] International Labour Organization. Main statistics (annual)—Economically active population. Available at: http://laborsta.ilo.org/applv8/data/c1e.html, 2014.

[30] Udry C. Child labor. Yale University Economic Growth Center 2003 June;Discussion Paper no. 856:21p.

[31] Unicef. Child labour. Available at: http://data.unicef.org/child-protection/child-labour, 2014.

[32] Bloom DE, McKinnon R. The design and implementation of public pension systems in developing countries: Issues and options. Harvard Program on the Global Demography og Aging Working Paper Series 2013 May;PGDA Working Paper No. 102:pg 2.

[33] International Labour Organization. World Social Security Report 2010–11: Providing coverage in times of crisis and beyond. 2010:pg 32; pg 46.

[34] Brouwer WB, Koopmanschap MA, Rutten FF. Productivity costs measurement through quality of life? A response to the recommendation of the Washington Panel. Health Econ 1997 May-Jun;6(3):253–259. 9226143 10.1002/(sici)1099-1050(199705)6:3<253::aid-hec266>3.0.co;2-6

[35] Nyman JA. Productivity costs revisited: towards a new US policy. Health Econ 2012;21(12):1387–1401.

[36] Lenk EJ, Redekop WK, Luyendijk M, Rijnsburger AJ, Severens JL. Productivity Loss Related to Neglected Tropical Diseases Eligible for Preventive Chemotherapy: A Systematic Literature Review. PLoS Negl Trop Dis 2016 2 18;10(2):e0004397 10.1371/journal.pntd.0004397 26890487 PMC4758606

[37] Frick KD, Foster A, Pizzarello L. The magnitude and cost of global blindness: An increasing problem that can be alleviated. Evid -Based Eye Care 2003;4:170–171. 10.1016/s0002-9394(02)02110-4 12654362

[38] Frick KD, Basilion EV, Hanson CL, Colchero AM, Luo B, Brown MM. Estimating the burden and economic impact of trachomatous visual loss. Evid -Based Eye Care 2003;4:210–211. 10.1076/opep.10.2.121.13899 12660860

[39] Murray CJ, Ezzati M, Flaxman AD, Lim S, Lozano R, Michaud C, et al GBD 2010: design, definitions, and metrics. The Lancet; 380(9859):2063–2066. 10.1016/S0140-6736(12)61899-6 23245602

[40] Chu BK, Hooper PJ, Bradley MH, McFarland DA, Ottesen EA. The Economic Benefits Resulting from the First 8 Years of the Global Programme to Eliminate Lymphatic Filariasis (2000–2007). Plos Neglected Tropical Diseases 2010 6;4. 10.1371/journal.pntd.0000708 PMC2879371 20532228

[41] Drummond M, Sculpher M, Torrance B, O'brien B, Stoddart G. Methods for the Economic Evaluation of Health Care Programmes. third ed. Oxford: Oxford University Press; 2005.

[42] The World Bank. World Development Indicators. Available at: http://data.worldbank.org/data-catalog/world-development-indicators, 2014.

[43] Babu BV, Nayak AN, Dhal K, Acharya AS, Jangid PK, Mallick G. The economic loss due to treatment costs and work loss to individuals with chronic lymphatic filariasis in rural communities of Orissa, India. Acta Trop 2002 4;82(1):31–38. 11904101 10.1016/s0001-706x(02)00030-x

[44] Babu BV, Nayak AN. Treatment costs and work time loss due to episodic adenolymphangitis in lymphatic filariasis patients in rural communities of Orissa, India. Trop Med Int Health 2003;8:1102–1109. 14641845 10.1046/j.1360-2276.2003.01146.x

[45] Budge PJ, Little KM, Mues KE, Kennedy ED, Prakash A, Rout J, et al Impact of Community-Based Lymphedema Management on Perceived Disability among Patients with Lymphatic Filariasis in Orissa State, India. PLoS Negl Trop Dis 2013;7. 10.1371/journal.pntd.0002100 PMC3597476 23516648

[46] Chandrasena TN, Premaratna R, De Silva NR. Lymphoedema management knowledge and practices among patients attending filariasis morbidity control clinics in Gampaha District, Sri Lanka. Filaria J 2004 8 3;3:6 10.1186/1475-2883-3-6 15287989 PMC514715

[47] Gasarasi DB, Premji ZG, Mujinja PG, Mpembeni R. Acute adenolymphangitis due to bancroftian filariasis in Rufiji district, south east Tanzania. Acta Trop 2000 2 25;75(1):19–28. 10708003 10.1016/s0001-706x(99)00090-x

[48] Ramaiah KD, Vijay Kumar KN, Ramu K, Pani SP, Das PK. Functional impairment caused by lymphatic filariasis in rural areas of South India. Trop Med Int Health 1997;2:832–838. 9315041 10.1046/j.1365-3156.1997.d01-406.x

[49] Ramaiah KD, Guyatt H, Ramu K, Vanamail P, Pani SP, Das PK. Treatment costs and loss of work time to individuals with chronic lymphatic filariasis in rural communities in south India. Trop Med Int Health 1999;4:19–25. 10203169 10.1046/j.1365-3156.1999.00351.x

[50] Ramaiah KD, Radhamani MP, John KR, Evans DB, Guyatt H, Joseph A, et al The impact of lymphatic filariasis on labour inputs in southern India: Results of a multi-site study. Ann Trop Med Parasitol 2000;94:353–364. 10945045 10.1080/00034983.2000.11813550

[51] Babu BV, Swain BK, Rath K. Impact of chronic lymphatic filariasis on quantity and quality of productive work among weavers in an endemic village from India. Trop Med Int Health 2006 5;11(5):712–717. 10.1111/j.1365-3156.2006.01617.x 16640624

[52] Ramaiah KD, Ramu K, Guyatt H, Vijar Kumar KN, Pani SP. Direct and indirect costs of the acute form of lymphatic filariasis to households in rural areas of tamil nadu, south india. Trop Med Int Health 1998;3:108–115. 9537272 10.1046/j.1365-3156.1998.00208.x

[53] Evans TG. Socioeconomic consequences of blinding onchocerciasis in west Africa. Bull World Health Organ 1995;73(4):495–506. 7554022 PMC2486790

[54] Fenwick A, Figenschou B. The effect of Schistosoma mansoni infection of the productivity of cane cutters on a sugar estate in Tanzania. Bull World Health Organ 1972;47(5):567–572. 4540675 PMC2480845

[55] Kim A, Tandon A, Hailu A. Health and Labor Productivity: The Economic Impact of Onchocercial Skin Disease. 1997.

[56] Umeh JC, Amali O, Umeh EU. Impact of urinary schistosomiasis on rural land use: empirical evidence from Nigeria. Soc Sci Med 2001 1;52:293–303. 11144785 10.1016/s0277-9536(00)00134-9

[57] Palmer K. Management of haematemesis and melaena. Postgrad Med J 2004 7;80(945):399–404. 10.1136/pgmj.2003.017558 15254304 PMC1743041

[58] Ducóns García J, Aranguren García FJ. Protocolo diagnóstico y valoración pronóstica de la hematemesis. Medicine—Programa de Formación Médica Continuada Acreditado 2012 2;11(2):123–126.

[59] Basta SS, Soekirman, Karyadi D, Scrimshaw NS. Iron deficiency anemia and the productivity of adult males in Indonesia. Am J Clin Nutr 1979;32:916–925. 107787 10.1093/ajcn/32.4.916

[60] Hunt JM. Reversing productivity losses from iron deficiency: the economic case. J Nutr 2002 4;132(4 Suppl):794S–801S. 11925484 10.1093/jn/132.4.794S

[61] Ross JS, Horton S. Economic Consequences of Iron Deficiency. Micronutrient Initiative 1998:1–42.

[62] Selvaratnam RR, de Silva LD, Pathmeswaran A, de Silva NR. Nutritional status and productivity of Sri Lankan tea pluckers. Ceylon Med J 2003;48:114–118. 15125401 10.4038/cmj.v48i4.3326

[63] Gilgen D, Mascie‐Taylor C, Rosetta L. Intestinal helminth infections, anaemia and labour productivity of female tea pluckers in Bangladesh. Tropical medicine & international health 2001;6(6):449–457. 11422959 10.1046/j.1365-3156.2001.00729.x

[64] Wolgemuth JC, Latham MC, Hall A, Chesher A, Crompton DW. Worker productivity and the nutritional status of Kenyan road construction laborers. Am J Clin Nutr 1982 7;36(1):68–78. 7091036 10.1093/ajcn/36.1.68

[65] Tanner S, Rosinger A, Leonard WR, Reyes-Garcia V. Health and adult productivity: The relation between adult nutrition, helminths, and agricultural, hunting, and fishing yields in the Bolivian Amazon. Am J Human Biol 2013;25:123–130. 23180686 10.1002/ajhb.22350

[66] World Health Organization. Treatment and control of onchocerciasis. Available at: http://www.who.int/apoc/onchocerciasis/control/en/. Accessed September/24, 2014.

[67] Bethony J, Brooker S, Albonico M, Geiger SM, Loukas A, Diemert D, et al Soil-transmitted helminth infections: ascariasis, trichuriasis, and hookworm. Lancet 2006;367:1521–1532. 10.1016/S0140-6736(06)68653-4 16679166

[68] Gryseels B, Polman K, Clerinx J, Kestens L. Human schistosomiasis. Lancet 2006 9 23;368(9541):1106–1118. 10.1016/S0140-6736(06)69440-3 16997665

[69] Rajak SN, Habtamu E, Weiss HA, Bedri A, Zerihun M, Gebre T, et al Why do people not attend for treatment for trachomatous trichiasis in Ethiopia? A study of barriers to surgery. PLoS neglected tropical diseases 2012;6(8):e1766 10.1371/journal.pntd.0001766 22953007 PMC3429389

[70] Bowman NM, Kawai V, Levy MZ, Del Carpio J. G. C., Cabrera L, Delgado F, et al Chagas disease transmission in periurban communities of Arequipa, Peru. Clin Infect Dis 2008;46:1822–1828. 10.1086/588299 18462104

[71] Bowman RJC, Faal H, Jatta B, Myatt M, Foster A, Johnson GJ, et al Longitudinal study of trachomatous trichiasis in The Gambia: Barriers to acceptance of surgery. Invest Ophthalmol Vis Sci 2002;43:936–940. 11923231

[72] Bowman RJC, Sey Soma O, Alexander N, Milligan P, Rowley J, Faal H, et al Should trichiasis surgery be offered in the village? A community randomised trial of village vs. health centre-based surgery. Trop Med Int Health 2000;5:528–533. 10995093 10.1046/j.1365-3156.2000.00605.x

[73] Melese M, Alemayehu W, Friedlander E, Courtright P. Indirect costs associated with accessing eye care services as a barrier to service use in Ethiopia. Trop Med Int Health 2004 3;9:426–431. 14996373 10.1111/j.1365-3156.2004.01205.x

[74] Frick KD, Keuffel EL, Bowman RJ. Epidemiological, demographic, and economic analyses: measurement of the value of trichiasis surgery in The Gambia. Ophthalmic Epidemiol 2001 7;8:191–201. 11471088 10.1076/opep.8.2.191.4163

[75] Ahorlu CK, Dunyo SK, Asamoah G, Simonsen PE. Consequences of hydrocele and the benefits of hydrocelectomy: A qualitative study in lymphatic filariasis endemic communities on the coast of Ghana. Acta Trop 2001;80:215–221. 11700178 10.1016/s0001-706x(01)00159-0

[76] Cantey PT, Richard S, Dorkenoo A, Sodahlon Y, Mathieu E. Patient treatment costs for management of lymphedema and acute attacks in Togo. Am J Trop Med Hyg 2009;81:94.19556573

[77] Gyapong JO. The economic burden of lymphatic filariasis in northern Ghana. Ann Trop Med Parasitol 1996;90:39–48. 8729626 10.1080/00034983.1996.11813024

[78] Gyapong M, Gyapong J, Weiss M, Tanner M. The burden of hydrocele on men in Northern Ghana. Acta Trop 2000;77:287–294. 11114391 10.1016/s0001-706x(00)00145-5

[79] Gyapong M. Socio-Cultural Aspects of Lymphatic Filariasis and The Role of Communities in its Control in Ghana. Basel: University of Basel; 6 2000.

[80] Nanda B, Krishnamoorthy K. Treatment seeking behaviour and costs due to acute and chronic forms of lymphatic filariasis in urban areas in south India. Trop Med Int Health 2003;8:56–59. 12535251 10.1046/j.1365-3156.2003.00962.x

[81] Perera M, Whitehead M, Molyneux D, Weerasooriya M, Gunatilleke G. Neglected patients with a neglected disease? A qualitative study of lymphatic filariasis. PLoS Negl Trop Dis 2007;1. 10.1371/journal.pntd.0000128 PMC2100378 18060080

[82] Ramaiah K, Guyatt H, Ramu K, Vanamail P, Pani S, Das P. Treatment costs and loss of work time to individuals with chronic lymphatic filariasis in rural communities in south India. Tropical Medicine & International Health 1999;4(1):19–25. 10203169 10.1046/j.1365-3156.1999.00351.x

[83] Taylor MJ, Hoerauf A, Bockarie M. Lymphatic filariasis and onchocerciasis. Lancet 2010 10 2;376(9747):1175–1185. 10.1016/S0140-6736(10)60586-7 20739055

[84] Fitzpatrick C, Nwankwo U, de Vlas S, Bundy D. An Investment Case for Ending Neglected Tropical Diseases In: Holmes KK, Bertozzi S, Boolm B, Jha P, Nugent R, editors. *Disease Control Priorities*, *Third Edition*. Volume 6: *Major Infectious Diseases*. Third Edition ed. Washington DC: World Bank; Forthcoming 2016.

[85] World Health Organization. Definition of region groupings. Available at: http://www.who.int/healthinfo/global_burden_disease/definition_regions/en/. Accessed March/22, 2015.

[86] Koopmanschap MA, Rutten FF, van Ineveld BM, Van Roijen L. The friction cost method for measuring indirect costs of disease. J Health Econ 1995;14(2):171–189. 10154656 10.1016/0167-6296(94)00044-5

[87] Wogu M.D. OCE. Prevalence and socio-economic effects of onchocerciasis in Okpuje, Owan West Local Government Area, Edo State, Nigeria. International Journal of Biomedical and Health Sciences 2008 9 30;4(3):113–119.

[88] Gilgen D, Mascie‐Taylor C, Rosetta L. Intestinal helminth infections, anaemia and labour productivity of female tea pluckers in Bangladesh. Tropical medicine & international health 2001;6(6):449–457. 11422959 10.1046/j.1365-3156.2001.00729.x

[89] Brown LE. Plan B 4.0—Mobilizing to Save Civilization. New York, USA: W. W. Norton & Company; 2009.

[90] Food and Agriculture Organization of the United Nations—FAO. Concepts and impacts of conservation agriculture. Available at: http://www.fao.org/docrep/003/y1730e/y1730e03.htm. Accessed July/30, 2015.

[91] Food and Agriculture Organization of the United Nations—FAO. Land husbandry—Components and strategy. Available at: http://www.fao.org/docrep/t1765e/t1765e07.htm. Accessed July, 30, 2015.

[92] Oladepo O, Brieger WR, Otusanya S, Kale OO, Offiong S, Titiloye M. Farm land size and onchocerciasis status of peasant farmers in south- western Nigeria. Trop Med Int Health 1997;2:334–340. 9171841 10.1111/j.1365-3156.1997.tb00148.x

[93] Bleakley H. Disease and development: Evidence from the American South. Journal of the European Economic Association 2003;1:376–386.

[94] Bundy DAP, Kremer M, Bleakley H, Jukes MCH, Miguel E. Deworming and Development: Asking the Right Questions, Asking the Questions Right. PLoS Negl Trop Dis 2009;3(1):e362– 10.1371/journal.pntd.0000362 19172186 PMC2627944

[95] Molyneux DH, Malecela MN. Neglected tropical diseases and the millenium development goals: why the "other diseases" matter: reality versus rhetoric. Parasit Vectors 2011 12 13;4:234– 10.1186/1756-3305-4-234 22166580 PMC3271994

[96] Baird S, Hicks JH, Kremer M, Miguel E. Worms At Work: Long-Run Impacts Of Child Health Gains. Working Paper 2012:71 pgs. 10.1093/qje/qjw022 PMC5094294 27818531

[97] Litt E, Baker MC, Molyneux D. Neglected tropical diseases and mental health: a perspective on comorbidity. Trends Parasitol 2012 5;28(5):195–201. 10.1016/j.pt.2012.03.001 22475459

[98] Jacob-Tacken KH, Koopmanschap MA, Meerding WJ, Severens JL. Correcting for compensating mechanisms related to productivity costs in economic evaluations of health care programmes. Health Econ 2005 5;14(5):435–443. 10.1002/hec.948 15497201

[99] Pauly MV, Nicholson S, Xu J, Polsky D, Danzon PM, Murray JF, et al A general model of the impact of absenteeism on employers and employees. Health Econ 2002 4;11(3):221–231. 11921319 10.1002/hec.648

[100] Knies S, Boonen A, Candel MJ, Evers SM, Severens JL. Compensation mechanisms for lost productivity: a comparison between four European countries. Value Health 2013 Jul-Aug;16(5):740–744. 10.1016/j.jval.2013.03.1624 23947966

[101] Virtanen M, Heikkila K, Jokela M, Ferrie JE, Batty GD, Vahtera J, et al Long working hours and coronary heart disease: a systematic review and meta-analysis. Am J Epidemiol 2012 10 1;176(7):586–596. 10.1093/aje/kws139 22952309 PMC3458589

[102] Virtanen M, Ferrie JE, Singh-Manoux A, Shipley MJ, Vahtera J, Marmot MG, et al Overtime work and incident coronary heart disease: the Whitehall II prospective cohort study. Eur Heart J 2010 7;31(14):1737–1744. 10.1093/eurheartj/ehq124 20460389 PMC2903713

[103] Virtanen M, Singh-Manoux A, Ferrie JE, Gimeno D, Marmot MG, Elovainio M, et al Long working hours and cognitive function: the Whitehall II Study. Am J Epidemiol 2009 3 1;169(5):596–605. 10.1093/aje/kwn382 19126590 PMC2727184

[104] Zheng Q, Chen Y, Zhang H, Chen J, Zhou X. The control of hookworm infection in China. Parasit Vectors 2009;2(1):44 10.1186/1756-3305-2-44 19775473 PMC2760515

[105] Fenwick A, Figenschou BH. The effect of Schistosoma mansoni infection of the productivity of cane cutters on a sugar estate in Tanzania. Bull World Health Organ 1972;47:567–572. 4540675 PMC2480845

[106] Audibert M, Etard J. Impact of schistosomiasis on rice output and farm inputs in Mali. Journal of African Economies 1998 2013/11/15;7:185–207.

[107] Ramaiah KD, Das PK, Michael E, Guyatt HL. The economic burden of lymphatic filariasis in India. Parasitol Today (Regul Ed) 2000;16:251–253. 10827432 10.1016/s0169-4758(00)01643-4

[108] Li R, Chen X, Yan H, Deurenberg P, Garby L, Hautvast JG. Functional consequences of iron supplementation in iron-deficient female cotton mill workers in Beijing, China. Am J Clin Nutr 1994 4;59(4):908–13. 8147338 10.1093/ajcn/59.4.908

[109] Edgerton VR, Gardner GW, Ohira Y, Gunawardena KA, Senewiratne B. Iron-deficiency anaemia and its effect on worker productivity and activity patterns. Br Med J 1979 12 15;2(6204):1546–1549. 534861 10.1136/bmj.2.6204.1546 PMC1597434

[110] Gardner GW, Edgerton VR, Senewiratne B, Barnard RJ, Ohira Y. Physical work capacity and metabolic stress in subjects with iron deficiency anemia. Am J Clin Nutr 1977 6;30(6):910–917. 868783 10.1093/ajcn/30.6.910

[111] Smith JL, Brooker S. Impact of hookworm infection and deworming on anaemia in non-pregnant populations: a systematic review. Tropical Medicine & International Health 2010;15(7):776–795. 20500563 10.1111/j.1365-3156.2010.02542.x PMC2916221

[112] International Labour Organization. Minimum nominal monthly wage (Local currency). Available at: http://www.ilo.org/ilostat/faces/help_home/data_by_subject/subject-details/indicator-details-by-subject?subject=EAR&indicator=EAR_INEE_NOC_NB&datasetCode=GWR&collectionCode=GWR&_afrLoop=1326635868954534#%40%3Findicator%3DEAR_INEE_NOC_NB%26subject%3DEAR%26_afrLoop%3D1326635868954534%26datasetCode%3DGWR%26collectionCode%3DGWR%26_adf.ctrl-state%3Dmoofax50c_250. Accessed March/03, 2015.

[113] The World Bank. PPP conversion factor (GDP) to market exchange rate ratio. Available at: http://data.worldbank.org/indicator/PA.NUS.PPPC.RF. Accessed July/18, 2014.

